# Population pharmacokinetics and individualized dosing of vancomycin for critically ill patients receiving continuous renal replacement therapy: the role of residual diuresis

**DOI:** 10.3389/fphar.2023.1298397

**Published:** 2023-12-29

**Authors:** Zhenwei Yu, Jieqiong Liu, Haitao Yu, Ling Zhou, Jianping Zhu, Gang Liang, Yi Yang, Ying Zheng, Yun Han, Junjun Xu, Gang Han, Lingyan Yu, Yuhua Zhao

**Affiliations:** ^1^ Sir Run Run Shaw Hospital, Zhejiang University School of Medicine, Hangzhou, China; ^2^ Research Center for Clinical Pharmacy, Zhejiang University, Hangzhou, China; ^3^ The 903rd Hospital of PLA Joint Logistic Support Force, Hangzhou, China; ^4^ Zhejiang Zhoushan Hospital, Zhoushan, China; ^5^ College of Pharmaceutical Science, Zhejiang University, Hangzhou, China; ^6^ The Second Affiliated Hospital, Zhejiang University School of Medicine, Hangzhou, China; ^7^ Affiliated Xiaoshan Hospital, Hangzhou Normal University, Hangzhou, China

**Keywords:** vancomycin, residual renal function, continuous renal replacement therapy, critically ill, population pharmacokinetics

## Abstract

**Background:** Vancomycin dosing is difficult in critically ill patients receiving continuous renal replacement therapy (CRRT). Previous population pharmacokinetic (PopPK) models seldom consider the effect of residual diuresis, a significant factor of elimination, and thus have poor external utility. This study aimed to build a PopPK model of vancomycin that incorporates daily urine volume to better describe the elimination of vancomycin in these patients.

**Methods:** We performed a multicenter retrospective study that included critically ill patients who received intermittent intravenous vancomycin and CRRT. The PopPK model was developed using the NONMEM program. Goodness-of-fit plots and bootstrap analysis were employed to evaluate the final model. Monte Carlo simulation was performed to explore the optimal dosage regimen with a target area under the curve of ≥400 mg/L h and 400–600 mg/L h.

**Results:** Overall, 113 observations available from 71 patients were included in the PopPK model. The pharmacokinetics could be well illustrated by a one-compartment model with first-order elimination, with the 24-h urine volume as a significant covariate of clearance. The final typical clearance was 1.05 L/h, and the mean volume of distribution was 69.0 L. For patients with anuria or oliguria, a maintenance dosage regimen of 750 mg q12h is recommended.

**Conclusion:** Vancomycin pharmacokinetics in critically ill patients receiving CRRT were well described by the developed PopPK model, which incorporates 24-h urine volume as a covariate. This study will help to better understand vancomycin elimination and benefit precision dosing in these patients.

## Introduction

Vancomycin is commonly used for severe infections caused by gram-positive bacteria, including methicillin-resistant *Staphylococcus aureus* (MRSA) (Levine, 2006; Tong et al., 2015; Brown et al., 2021). It has a narrow therapeutic window and thus needs precise personalized dosing to achieve maximum antibacterial efficacy and minimum toxicity (Rybak et al., 2020). Vancomycin is mainly eliminated through the kidney; therefore, the dose should be adjusted based on renal function (Aljutayli et al., 2020). Acute kidney injury (AKI) is prevalent in critically ill patients, and continuous renal replacement therapy (CRRT) is an important life-support technique that is increasingly being applied to these patients (Tandukar and Palevsky, 2019; Rybak et al., 2020). Vancomycin pharmacokinetics (PK) are altered in critically ill patients, and CRRT can generate complex factors affecting drug exposure and increase the PK variability of vancomycin; therefore, conventional dosing often fails to achieve therapeutic goals in patients receiving CRRT (Roberts et al., 2012; Li et al., 2019; Roberts et al., 2020).

Due to the heterogeneity of CRRT patients, the Chinese and Japanese guidelines cannot recommend an initial vancomycin dosing regimen for these patients (He et al., 2020; Matsumoto et al., 2022). The American Society of Health-System Pharmacists (ASHP) 2020 guideline recommends a therapeutic target of 400–600 AUC/MIC and a maintenance dose of 7.5–10 mg/kg every 12 h for CRRT patients with effluent rates of 20–25 mL/kg/h (Rybak et al., 2020). However, the recommendations lack solid evidence. Thus, it is challenging for physicians to administer vancomycin to these patients.

Several studies have investigated the population pharmacokinetics (PopPK) and optimal dosing regimens of vancomycin in these patients, but there is still controversy over these results (Wang et al., 2021; Chen et al., 2022; Wang et al., 2023). Most of these studies focused only on the influence of the characteristics of CRRT, including CRRT intensity or type, on the elimination of vancomycin (Claisse et al., 2019; Chen et al., 2022). Vancomycin can be significantly eliminated by CRRT (Chaijamorn et al., 2011; Li et al., 2019). However, the total drug clearance in these patients was calculated as the sum of the CRRT-induced clearance and non-CRRT clearance (Claisse et al., 2019). Residual renal function, the main route of non-CRRT elimination, cannot be neglected in most circumstances (Ulldemolins et al., 2015; Barrasa et al., 2019; Roberts et al., 2020). However, there have been only a few models for CRRT patients in which residual renal function is taken into account (Oda et al., 2020). This could be an important reason why previous PopPK models had poor external utility (Cunio et al., 2020).

Notably, the calculation of residual renal function is extremely complex and requires the collection of 24-h urine volume, urine creatinine, pre- and postdialysis serum creatinine, serum β-trace protein, and β2-microglobulin, which limits its clinical application. Thus, we aimed to identify a more convenient method, and residual diuresis seems to be a more parsimonious indicator of residual renal function (Daugirdas et al., 2015; Wong et al., 2016; Assimon and Flythe, 2020). In this study, we developed a PopPK model that described the elimination of vancomycin by CRRT and residual diuresis, which would be helpful for better understanding the mechanism of vancomycin elimination and dose optimization in patients receiving CRRT.

## Methods

### Study design and ethics approval

This was a multicenter, retrospective study. Ethics approval was obtained from the ethics committee of Sir Run Run Shaw Hospital, School of Medicine, Zhejiang University (reference number 2022-0259). The requirement for obtaining informed consent from the patients was waived due to the retrospective nature of the study.

### Patient inclusion criteria

We enrolled ICU patients who received intermittent intravenous vancomycin and CRRT from January 2019 to October 2022 at 4 centers (Sir Run Run Shaw Hospital, School of Medicine, Zhejiang University; Affiliated Xiaoshan Hospital, Hangzhou Normal University; Second Affiliated Hospital, School of Medicine, Zhejiang University; Zhejiang Zhoushan Hospital). The patients’ demographic data and individual laboratory parameters were collected from the hospital information system as described in our previous study (Yu et al., 2023). Briefly, the following data were recorded: age, sex, body weight (BW), height, body mass index (BMI), infection site, infection pathogen, 24-h urine volume (UV), type of CRRT, and other related laboratory test results. Moreover, the dosage regimen of vancomycin, the dosing time of vancomycin and the sampling time of plasma for vancomycin concentration determination were recorded. The vancomycin concentrations, which were determined by LC-MS/MS methods at all the centers, were also collected. Patients who met the following criteria were excluded: i) were aged <18 years; ii) lacked sufficient data (lack of BW, height, dosing time, or laboratory examination data during vancomycin treatment); iii) did not receive CRRT continuously during vancomycin treatment; and iv) whose plasma vancomycin concentration was determined more than 48 h after the last dose.

### PK analyses and PopPK modeling

PK analysis was performed using the nonlinear mixed-effects modeling program NONMEM (version 7.5.0, ICON, Ellicott City, MD, United States) and PDxPop (version 5.3.1, ICON, Gaithersburg, MD, United States). The graphical visualizations were performed with the R program (version 4.2.3; https://www.r-project.org/). The first-order conditional estimation with interaction method (FOCE-I) was used throughout the model development procedure.

Models were developed and evaluated based on the objective function value (OFV), Akaike information criterion (AIC) and goodness-of-fit plots. The one-and two-compartment models were tested as the base models. The intraindividual variability (residual error) was evaluated with additive, proportional, and combined error models.

The covariates were selected using a forward inclusion and backward elimination strategy. The screened covariates included demographic characteristics (age, sex, body weight, BMI), vancomycin dosing regimen (daily dose), renal functions (serum creatinine, blood urea nitrogen, UV), and type of CRRT (CVVH patients were defined as 1, and CVVHDF patients were defined as 2). Correlation analysis was performed before covariate model development. If the correlation coefficient of these two variables was >0.3, then one of the variables was selected for inclusion in the model based on whether it was clinically relevant or easily applied. The effects of continuous covariates were modeled using a median-normalized model, while the effects of categorical covariates were described by a power model. Covariates were included in the model based on the criteria of OFV requiring a decrease of 3.84 (*p* < 0.05) in forward inclusion and an increase of greater than 10.83 (*p* < 0.001) in backward elimination. Goodness-of-fit plots and bootstrap analysis were utilized to evaluate the final model and parameter estimates.

### Simulation and dosing regimen optimization

Monte Carlo simulation (MCS) was performed by utilizing the parameter estimates obtained from the final PopPK model to optimize the dosing regimen. The effect of the maintenance dose was simulated using doses of 500 mg q12h, 500 mg qd, 750 mg q12h, 1000 mg q12h, and 1500 mg q12h in different residual diuresis regimens (24-h urine volume with 0 mL, 100 mL, 500 mL, 1500 mL, 2000 mL, and 3,000 mL). The area under the curve (AUC) was calculated using the pkr package (version 0.1.3) in the R program with linear-up and linear-down methods. For the assessment of efficacy, the probability of target attainment (PTA) of an AUC threshold of ≥400 mg/L∙h and 400–600 mg/L∙h (assuming an MIC of 1 mg/L) was calculated. Optimal dosing regimens were chosen according to their capacity to attain a PTA of 90%.

## Results

### Patient inclusion and characteristics

**FIGURE 1:**
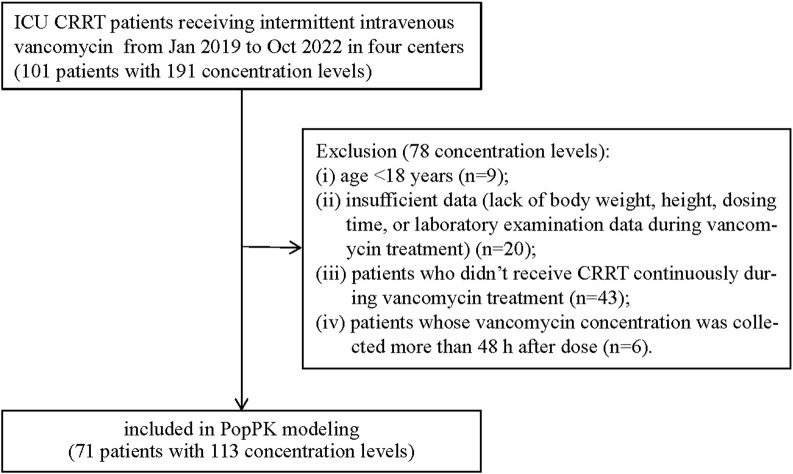
Flowchart of patient inclusion and exclusion criteria in this study. Abbreviations: ICU, intensive care unit; CRRT, continuous renal replacement therapy; PopPK, population pharmacokinetic.

In total, 191 trough concentrations were measured in 101 patients. The inclusion and exclusion criteria are presented in Figure 1. Finally, the remaining 113 trough concentrations available from 71 patients were eligible for inclusion in the development of the PopPK model. The demographic characteristics of the included patients are shown in Table 1.

**TABLE 1:** Demographic characteristics of the included patients.

Variable	Total (n = 71)	Min–Max
Sex, n (%)		
Male	44 (62.0%)	
Female	27 (38.0%)	
Age (years)	61.6 ± 14.6	24–87
Weight (kg)	64.2 ± 14.7	32.1–100
BMI (kg/m^2^)	23.3 ± 3.97	12.7–33.8
Infected site, n (%)		
Blood stream infection	18 (25.4%)	
Pulmonary infection	31 (43.7%)	
Intra-abdominal infection	17 (23.9%)	
Skin and soft tissue infection	3 (4.23%)	
Other or undefined	17 (23.9%)	
Pathogen, n (%)		
MRSA	6 (8.45%)	
MSSA	2 (2.82%)	
*Enterococcus*	10 (14.1%)	
CoNS	7 (9.86%)	
Other	16 (22.5%)	
undefined	35 (49.3%)	
Daily dose (mg/d)	1000 (500,2000)	500–3,000
Daily dose/weight (mg/kg/d)	15.4 (10.2,28.6)	5.26–50.0
Type of CRRT		
CVVH	40 (56.3%)	
CVVHDF	31 (43.7%)	
Laboratory data, median (IQR)		
SCr (μmol/L)	104 (62.0,202)	5.97–661
BUN (mmol/L)	7.80 (5.20,13.4)	0.93–45.2
24-h urine volume (mL)	160 (7.00,780)	0.00–6,220
≤100 mL	33 (46.5%)	
100–500 mL	12 (16.9%)	
≥2,500 mL	6 (8.45%)	
others	20 (28.2%)	

All renal function indices, as well as 24-h urine volume, were measured on the day of concentration monitoring. All continuous data are presented as the mean ± standard deviation (with normally distributed data) or median and interquartile range (with nonnormally distributed data); and categorical data are presented as numbers and percentages.

Abbreviations: BMI, body mass index; MRSA, methicillin-resistant *Staphylococcus aureus*; MSSA, methicillin-sensitive *Staphylococcus aureus*; CoNS, coagulase-negative *Staphylococcus*; CRRT, continuous renal replacement therapy; CVVH, continuous venovenous hemofiltration; CVVHDF, continuous venovenous hemodiafiltration; SCr, serum creatinine; BUN, blood urea nitrogen; ALT, alanine aminotransferase; AST, aspartate aminotransferase; ALP, alkaline phosphatase; TBIL, total bilirubin; TP, total protein; ALB, albumin; WBC, white blood cell; RBC, red blood cell; PLT, platelet; IQR, interquartile range.

### PopPK model development

The vancomycin concentration data could be well illustrated by a one-compartment model with first-order elimination (800.588 and 803.682 for AIC in the one- and two-compartment models, respectively). An exponential model and a proportional model were used to describe the interindividual variability and residual variability, respectively. The results of the correlation analysis are displayed in Supplementary Figure S1. The final PopPK model and parameters are shown in Table 2, and the key progression of covariate screening is shown in Supplementary Table S1. In the final model, only the logarithmic 24-h urine volume was a significant covariate for clearance (CL), and no covariates resulted in a significant improvement for the volume of distribution (V).

**TABLE 2:** Final model estimation parameters and bootstrap analysis.

Parameter	Final model	Bootstrap analysis	
	Estimate [RSE (%)]	Median estimate [RSE (%)]	95%CI
CL (L/h)	1.05 (17.2)	1.07 (18.7)	0.721–1.53
V (L)	69.0 (6.61)	68.6 (6.87)	59.9–77.7
θ_UV-CL_	1.90 (16.5)	1.91 (18.4)	1.31–2.71
Intre-individual variability			
ω (%) for CL	12.1 (47.9)	11.5 (45.0)	2.45–22.0
Residual variability			
σ (%)	9.78 (29.1)	9.60 (28.4)	4.70–15.2

The success rate was calculated as 100% (1000/1000).

The final model was as follows: CL (L/h)=1.05*1.90^LOG(UV+10)/2.3^, V (L) = 69.0.

Intre-individual variability for V was not estimated.

Abbreviations: CL, typical apparent clearance; V, typical apparent volume distribution; θ_UV-CL_, 24-h urine volume as a covariate for CL; ω, interindividual variance for CL; σ, residual variability for proportional error; RSE, residual standard error; CI, confidence interval.

### Model validation and bootstrap analysis

**FIGURE 2:**
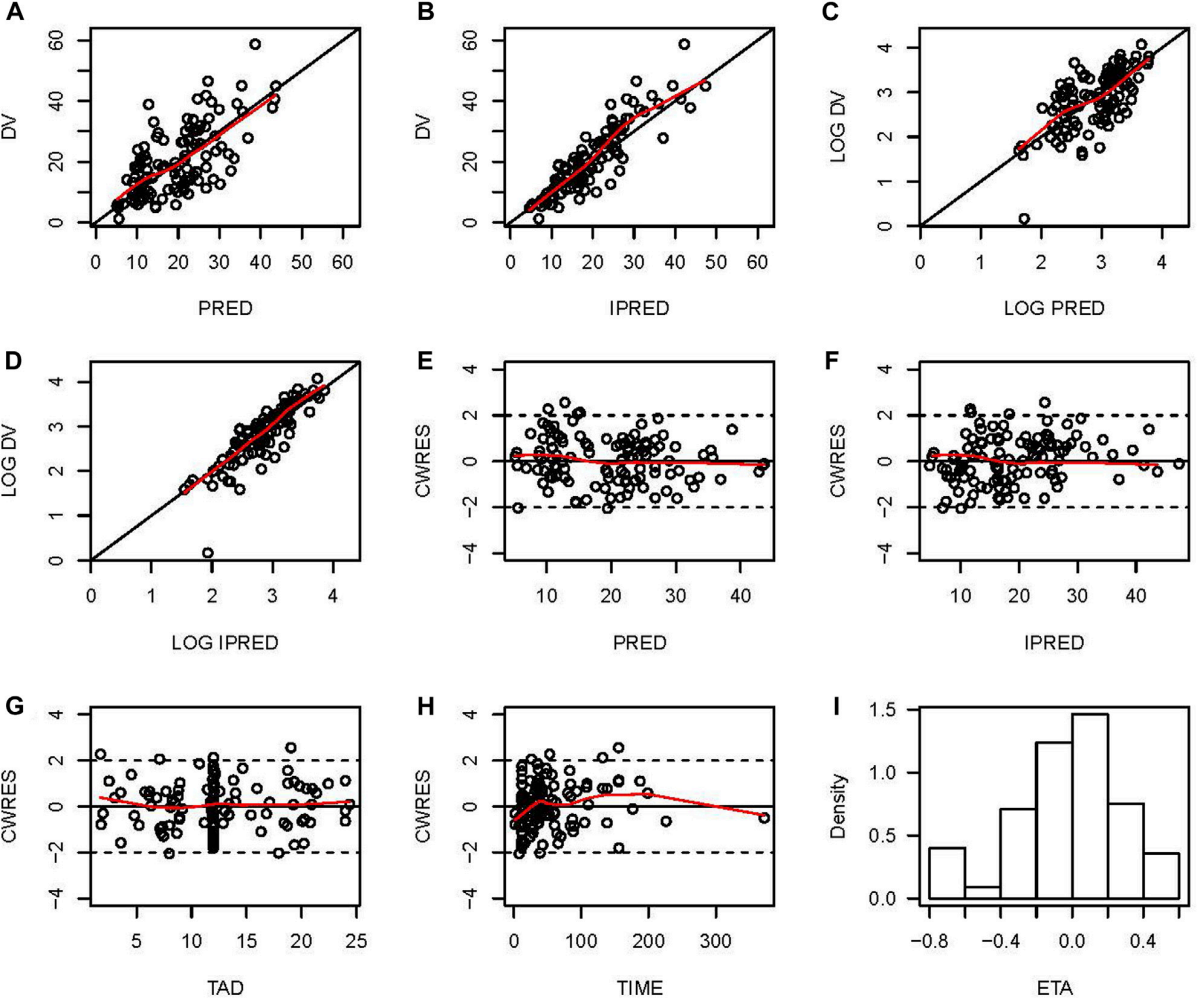
Goodness-of-fit plots for the final PopPK model. **(A)** Observed vancomycin concentrations (DV) *versus* population predictions (PRED); **(B)** DV *versus* individual predictions (IPRED); **(C)** Log-transformed DV *versus* Log-transformed PRED; **(D)** Log-transformed DV *versus* Log-transformed IPRED; **(E)** Conditional weighted residuals (CWRES) *versus* PRED; **(F)** Conditional individual weighted residuals (CIWRES) *versus* IPRED; **(G)** CWRES *versus* time after dose (TAD); **(H)** CWRES *versus* time; **(I)** distribution of ETA.

The final model was evaluated by goodness-of-fit plots and showed acceptable visual bias (Figure 2). The parameter estimates and between-subject variability from the final model and 1,000 bootstrap runs are presented in Table 2, which indicates the robustness of the final model.

### Simulation and dosing regimen optimization

**FIGURE 3:**
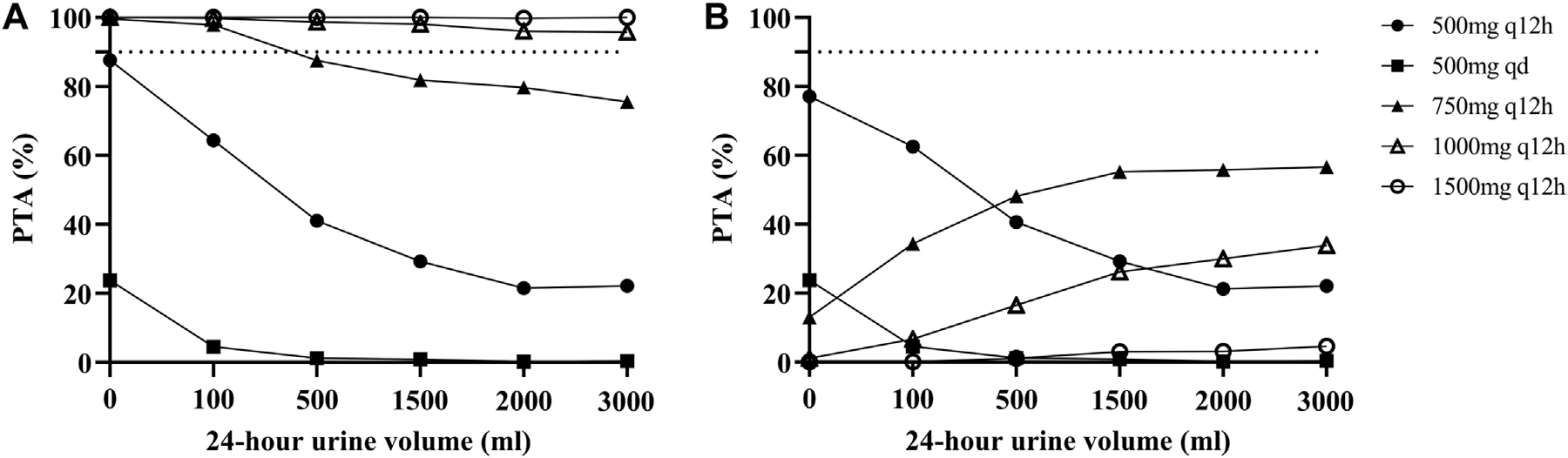
Probability of target attainment (PTA) for different dosage regimens of vancomycin according to the MCS. **(A)** PTA with an AUC ≥400 mg/L∙h; **(B)** PTA with an AUC between 400 and 600 mg/L∙h. The dashed lines indicate a PTA of 90%.

The PTAs for different dosage regimens for different residual diuresis of vancomycin for critically ill patients receiving CRRT according to the MCS are shown in Figure 3. For patients with anuria or oliguria, a maintenance dosage regimen of 750 mg q12h was suitable. However, the PTA decreased when the daily urine volume increased. A dose of 1000 mg q12h was associated with increased PTAs with an AUC ≥400 mg/L∙h under various residual diuresis. However, the probability of having an AUC ≥600 mg/L∙h was also greater. The AUC of the PTA regimens between 400 and 600 mg/L∙h was not satisfactory for all dosage regimens, which highlights the need for Bayesian forecasting of dosing and close TDM in these patients.

## Discussion

To the best of our knowledge, this was the first multicenter retrospective study to develop a vancomycin PopPK model in adult critically ill patients receiving CRRT that incorporated residual renal function as a covariate. Our study provides a new perspective on vancomycin pharmacokinetics in CRRT patients and highlights the importance of taking residual renal function into consideration when vancomycin is administered to these patients. The logarithmic 24-h urine volume, an indicator of residual renal function, was the unique covariate in the final model that had a significant effect on the clearance of vancomycin.

Due to the difficulty of calculating residual renal function in routine clinical practice, few PopPK models have incorporated this covariate (Daugirdas et al., 2015; Wong et al., 2016; Oda et al., 2020). The serum creatinine (SCr) and blood urea nitrogen (BUN) levels may not accurately reflect renal function in patients with AKI who are receiving CRRT. Thus, residual diuresis is an easy and costless way to evaluate residual renal function in ICU patients (Assimon and Flythe, 2020). A single-center retrospective cohort study showed that an increase in 24-h urine volume resulted in a decrease in vancomycin concentrations in patients on CVVHD according to multivariate regression (Quinn et al., 2022). The median 24-h urine volume in that study was only 15 mL, but it still significantly affected the vancomycin concentration. This highlighted the importance of residual renal function in vancomycin elimination and indicated the possibility of using urine volume as an indicator of residual renal function. Oda K et al. included reduced urine output (RUO) as a categorical covariate in a PopPK model of vancomycin, suggesting that patients with RUO had significantly lower non-CRRT clearance than patients without RUO (the mean clearance of non-CRRT was 2.12 L/h, which was reduced to 0.34 L/h when the urine output was <0.5 mL/kg/h) (Oda et al., 2020). However, it cannot reflect the complexity of residual renal function using a categorical indicator. Moreover, the sample size was small, and only 3 patients without RUO were included in that study (14 patients with RUO). In our study, 24-h urine volume was successfully incorporated as a continuous covariate of CL. We can see from the final model that when the 24-h urine volume was close to 0, the CL was the intrinsic clearance of CRRT. When the 24-h urine volume increased, residual renal function contributed to the total clearance of vancomycin. The developed model could simultaneously reflect the mechanism by which vancomycin can be eliminated in these patients by CRRT and residual renal function. This result would help us understand the pharmacokinetics of vancomycin, and it would benefit vancomycin dosing in patients with different residual diuresis.

Previous studies have concluded that CRRT-induced clearance is primarily related to CRRT intensity or type (DelDot et al., 2004; Covajes et al., 2013; Jamal et al., 2014; Wahby et al., 2021). However, there are also different views. Udy et al. reported that the intensity of CRRT was poorly correlated with CL (*r* ^2^ = 0.01) (Udy et al., 2013). Another previous study revealed that different CRRT modalities do not significantly affect the clearance of vancomycin, which supports the use of vancomycin without distinguishing the specific CRRT modality (Chen et al., 2022). We did not explore the effect of the intensity of CRRT on clearance in this study. Although the inclusion of the categorical covariate of CRRT modality decreased the OFV by 4.982, it was removed during the backward elimination process.

In addition, we performed MCS based on different 24-h urine volumes and selected the dose regimen with the highest PTA as the final recommended dose regimen. Finally, the PTAs suggested that the maintenance dose of 1000 mg q12h, which is the most commonly employed dose in practice, would have higher PTAs for an AUC ≥400 mg/L∙h under various residual diuresis regimens, and a dosage regimen of 750 mg q12h may be appropriate for patients with anuria or oliguria. This new vancomycin nomogram provides an evidence-based approach for individualizing vancomycin dosages. However, all regimens had unsatisfactory PTAs when using an AUC of 400–600 mg/L∙h as a target. This may be due to the high interindividual viability of these patients. This result also emphasized the importance of routine TDM for these patients.

This study has several limitations. Some bias may exist because of the retrospective, observational nature of the current study. Data on the CRRT parameter settings were not collected, and the actual intensity of CRRT was difficult to determine accurately. Most vancomycin concentrations are trough concentrations; thus, the interindividual viability of the distribution volume cannot be estimated. Finally, this model lacks a large number of patients for external validation, which is needed in the future.

## Conclusion

This was a multicenter retrospective study to develop a vancomycin PopPK model that incorporates residual diuresis in critically ill patients receiving CRRT. The vancomycin pharmacokinetics in this population were well described by the final model, with 24-h urine volume serving as a covariate on clearance. This study highlighted the importance of residual diuresis in the elimination of vancomycin and provided a new perspective on the pharmacokinetics of vancomycin, as well as other antibiotics, in patients receiving CRRT. This study provided important evidence for vancomycin dosage individualization in these populations.

## Data Availability

The original contributions presented in the study are included in the article/Supplementary Material, further inquiries can be directed to the corresponding authors.
