# Supplementary figures and images for: Population pharmacokinetics and individualized dosing of vancomycin for critically ill patients receiving continuous renal replacement therapy: the role of residual diuresis
Source: Front Pharmacol. 2023 Dec 29;14:1298397. doi: 10.3389/fphar.2023.1298397 (PMC10785304; doi:10.3389/fphar.2023.1298397)

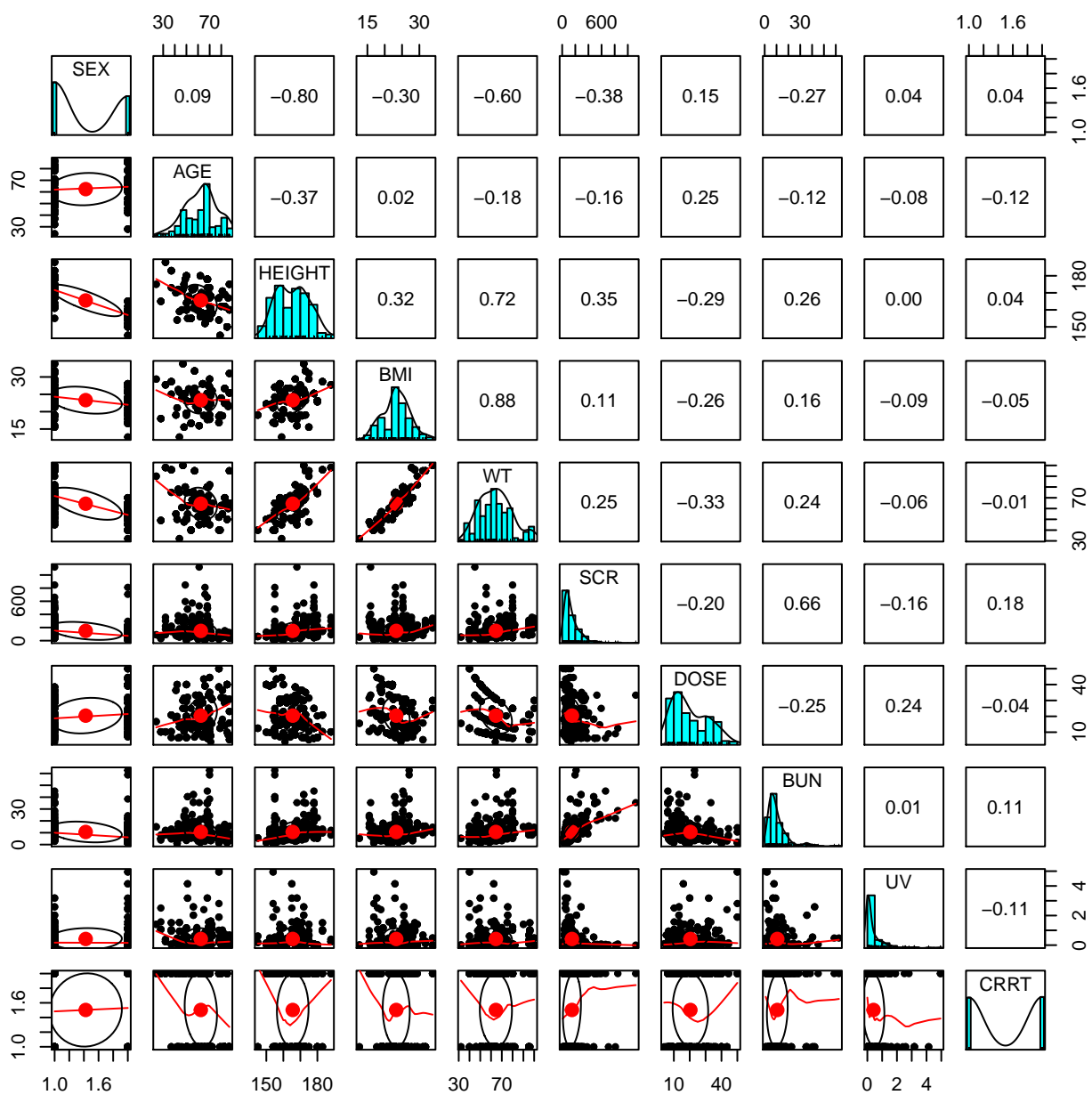

Fig. S1 Correlation matrix of covariates

Supplement: Supplementary file 1 [file DataSheet2.PDF]
