# Supplementary material for: Population pharmacokinetics and individualized dosing of vancomycin for critically ill patients receiving continuous renal replacement therapy: the role of residual diuresis
Source: Front Pharmacol. 2023 Dec 29;14:1298397. doi: 10.3389/fphar.2023.1298397 (PMC10785304; doi:10.3389/fphar.2023.1298397)
Supplement: Supplementary file 2 [file DataSheet1.PDF]

Table S1 Key process of covariate screening

Step	Model Description	Functional form	OFV	ΔOFV	p value
Forward inclusion					
1	Base model		585.366		
2	add UV on CL based on 1	power	569.226	-16.14	< 0.001
3	add SCr on CL based on 2	power	563.09	-6.136	< 0.05
4	add BMI on CL based on 3	linear	553.69	-9.4	< 0.01
5	add CRRT on CL based on 4	power	548.708	-4.982	< 0.05
6	add BW on V based on 1	power	583.931	-1.435	> 0.05
7	add BMI on V based on 1	power	582.687	-2.679	> 0.05
8	add SEX on V based on 1	power	585.343	-0.023	> 0.05
Backward elimination					
9	eliminate CRRT based on 5		553.685	4.977	> 0.001
10	eliminate BMI based on 9		563.09	9.405	> 0.001
11	eliminate SCR based on 10		569.226	6.136	> 0.001
12	eliminate UV based on 11		585.366	16.14	< 0.001

Abbreviations: OFV, objective function value; UV, 24-hour urine volume; SCr, serum creatinine; BMI, body mass index; CRRT, continuous renal replacement therapy; BW, body weight.
